# Supplementary figures and images for: Adults vs. neonates: Differentiation of functional connectivity between the basolateral amygdala and occipitotemporal cortex
Source: PLoS One. 2020 Oct 19;15(10):e0237204. doi: 10.1371/journal.pone.0237204 (PMC7571669; doi:10.1371/journal.pone.0237204)

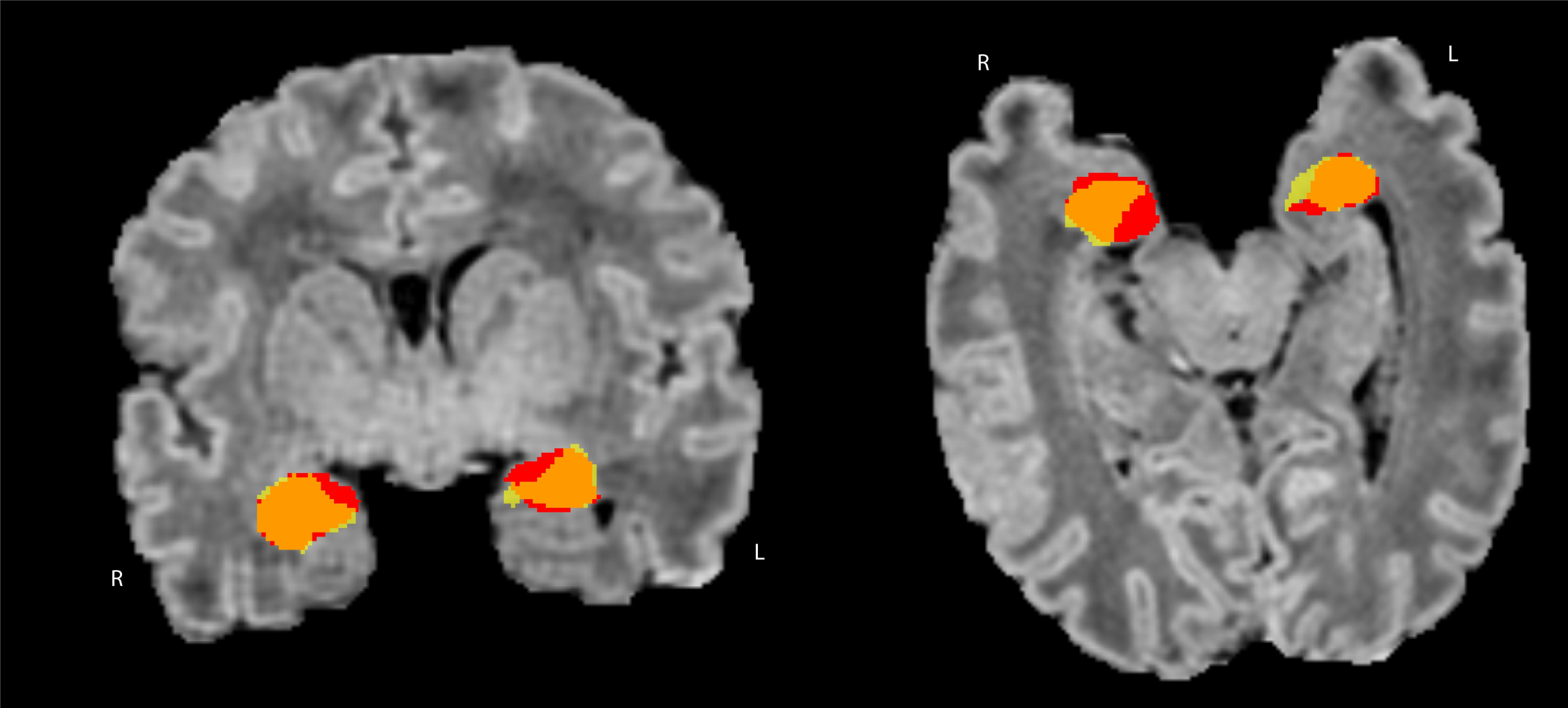

Supplement: S1 Fig — (left) Coronal and axial slices depicting the basolateral amygdala (yellow) as defined by automated segmentation (Saygin et al., 2017), overlaid on the whole amygdala as defined by dHCP’s DrawEm label (red). Overlap shown in orange (proportion overlap across all neonates: 0.76 ± 0.11) (TIF) [file pone.0237204.s001.tif]

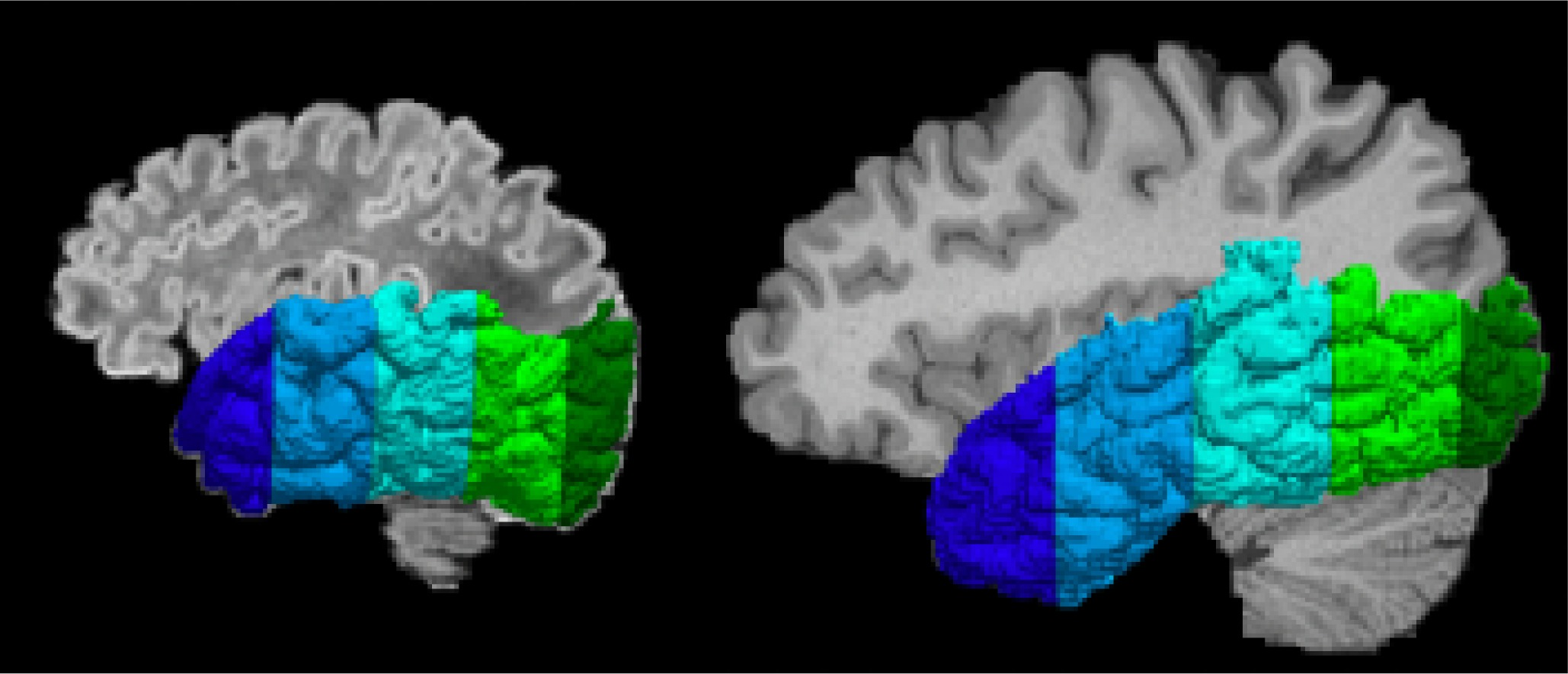

Supplement: S2 Fig — (left) neonate, (right) adult. Dark blue = OTC 5 (anterior), blue = OTC 4, light blue = OTC 3, lime green = OTC 2, dark green = OTC 1 (posterior). (TIF) [file pone.0237204.s002.tif]
