# Supplementary material for: Adults vs. neonates: Differentiation of functional connectivity between the basolateral amygdala and occipitotemporal cortex
Source: PLoS One. 2020 Oct 19;15(10):e0237204. doi: 10.1371/journal.pone.0237204 (PMC7571669; doi:10.1371/journal.pone.0237204)
Supplement: S1 Table — t-test results and corresponding p-values comparing mean connectivity between each OTC section, collapsed across adults and neonates. (DOCX) [file pone.0237204.s003.docx]

**S1 Table. OTC Connectivity Differences Collapsed Across Samples.**

| **OTC comparison** | ***t*** | ***p***_HB_ |
| --- | --- | --- |
| 5 – 4  5 – 3  5 – 2  5 – 1 | -4.438  -5.011  -7.406  -6.955 | 1.455 x 10^-4^  2.266 x 10^-5^  1.237 x 10^-9^  8.212 x 10^-9^ |
| 4 – 3  4 – 2  4 – 1 | -2.550  -5.074  -4.911 | 0.025  2.021 x 10^-5^  2.877 x 10^-5^ |
| 3 – 2  3 – 1 | -4.035  -3.926 | 5.005 x 10^-4^  5.506 x 10^-4^ |
| 2 – 1 | -2.058 | 0.043 |

t-test results and corresponding p-values comparing mean connectivity between each OTC section, collapsed across adults and neonates.

Note: p-values are Holm-Bonferroni corrected.
