# Supplementary material for: Adults vs. neonates: Differentiation of functional connectivity between the basolateral amygdala and occipitotemporal cortex
Source: PLoS One. 2020 Oct 19;15(10):e0237204. doi: 10.1371/journal.pone.0237204 (PMC7571669; doi:10.1371/journal.pone.0237204)
Supplement: S2 Table — t-test results and corresponding p-values comparing mean connectivity between each OTC section, separately for adults and neonates. (DOCX) [file pone.0237204.s004.docx]

**S2 Table. OTC Connectivity Differences Within Each Sample.**

| **Sample** | **OTC comparison** | ***t*** | ***p***_HB_ |
| --- | --- | --- | --- |
| *Adults* | 5 – 4  5 – 3  5 – 2  5 – 1 | -2.907  -3.633  -7.849  -8.426 | 0.012  0.003  1.357 x 10^-8^  2.581 x 10^-9^ |
|  | 4 – 3  4 – 2  4 – 1 | -1.995  -5.835  -6.748 | 0.053  4.393 x 10^-6^  3.812 x 10^-7^ |
|  | 3 – 2  3 – 1 | -6.056  -6.448 | 2.598 x 10^-6^  8.651 x 10^-7^ |
|  | 2 – 1 | -3.531 | 0.003 |
| *Neonates* | 5 – 4  5 – 3  5 – 2  5 – 1 | -3.327  -3.411  -3.198  -2.241 | 0.017  0.015  0.022  0.216 |
|  | 4 – 3  4 – 2  4 – 1 | -1.571  -1.186  -0.135 | 0.746  1.148  1.706 |
|  | 3 – 2  3 – 1 | 0.186  0.841 | 1.706  1.216 |
|  | 2 – 1 | 1.221 | 1.148 |

t-test results and corresponding p-values comparing mean connectivity between each OTC section, separately for adults and neonates.

Note: p-values are Holm-Bonferroni corrected within sample.
