# Supplementary material for: Adults vs. neonates: Differentiation of functional connectivity between the basolateral amygdala and occipitotemporal cortex
Source: PLoS One. 2020 Oct 19;15(10):e0237204. doi: 10.1371/journal.pone.0237204 (PMC7571669; doi:10.1371/journal.pone.0237204)
Supplement: S3 Table — t-test results and corresponding p-values comparing mean-centered connectivity between adults vs. neonates in each OTC section, from 5 (anterior) to 1 (posterior). See Fig 1C in main manuscript. (DOCX) [file pone.0237204.s005.docx]

**S3 Table. OTC Connectivity Differences Between Samples.**

| **OTC section** | ***t*** | ***p***_HB_ |
| --- | --- | --- |
| 5  4  3  2  1 | 3.813  4.397  3.744  -3.817  -5.923 | 8.064 x 10^-4^  1.373 x 10^-4^  5.458 x 10^-4^  8.064 x 10^-4^  4.042 x 10^-7^ |

t-test results and corresponding p-values comparing mean-centered connectivity between adults vs. neonates in each OTC section, from 5 (anterior) to 1 (posterior). See Fig 1C in main manuscript.

Note: p-values are Holm-Bonferroni corrected.
