# Supplementary material for: Adults vs. neonates: Differentiation of functional connectivity between the basolateral amygdala and occipitotemporal cortex
Source: PLoS One. 2020 Oct 19;15(10):e0237204. doi: 10.1371/journal.pone.0237204 (PMC7571669; doi:10.1371/journal.pone.0237204)
Supplement: S4 Table — t-test results and corresponding p-values comparing mean connectivity between each functional parcel in adults. (DOCX) [file pone.0237204.s006.docx]

**S4 Table. Connectivity Differences Between Functional Parcels in Adults.**

| **Parcel 1** | **Parcel 2** | ***t*** | ***p***_HB_ |
| --- | --- | --- | --- |
| A1 | STG | -5.683 | 8.144 x 10^-5^ |
|  | PPA  STS  PFS | -1.110  -3.721  -3.738 | 2.740  0.023  0.023 |
|  | EBA  LO  FFA  RSC | -4.146  -2.541  -3.787  1.497 | 0.008  0.424  0.021  1.996 |
|  | OFA  V1 | -1.763  3.237 | 1.457  0.084 |
| STG | PPA  STS  PFS | 4.430  2.130  1.543 | 0.004  0.909  2.096 |
|  | EBA  LO  FFA  RSC | 1.492  2.384  1.538  5.945 | 1.996  0.574  2.096  3.585 x 10^-5^ |
|  | OFA  V1 | 3.399  7.000 | 0.055  1.330 x 10^-6^ |
| PPA | STS  PFS | -2.722  -4.148 | 0.290  0.008 |
|  | EBA  LO  FFA  RSC | -4.233  -2.465  -3.833  3.783 | 0.006  0.492  0.019  0.021 |
|  | OFA  V1 | -1.007  6.138 | 2.882  2.001 x 10^-5^ |
| STS | PFS | -0.097 | 2.6611 |
|  | EBA  LO  FFA  RSC | -0.222  0.979  -0.261  4.350 | 3.978  2.882  4.485  0.005 |
|  | OFA  V1 | 2.181  6.498 | 0.847  6.443 x 10^-6^ |
| PFS | EBA  LO  FFA  RSC | -0.164  1.816  -0.324  5.263 | 3.303  1.463  4.713  2.836 x 10^-4^ |
|  | OFA  V1 | 3.114  8.455 | 0.114  1.539 x 10^-8^ |
| EBA | LO  FFA  RSC | 2.277  -0.080  5.542 | 0.709  1.847  1.230 x 10^-4^ |
|  | OFA  V1 | 3.080  9.027 | 0.121  2.825 x 10^-9^ |
| LO | FFA  RSC | -1.978  4.065 | 1.157  0.010 |
|  | OFA  V1 | 1.968  7.261 | 1.157  5.929 x 10^-7^ |
| FFA | RSC | 5.052 | 5.422 x 10^-4^ |
|  | OFA  V1 | 3.595  8.246 | 0.032  2.855 x 10^-8^ |
| RSC | OFA  V1 | -2.847  2.559 | 0.217  0.420 |
| OFA | V1 | 6.082 | 2.349 x 10^-5^ |

t-test results and corresponding p-values comparing mean connectivity between each functional parcel in adults.

Note: p-values are Holm-Bonferroni corrected across all comparisons. Lines divide parcels within the same OTC section.
