# Supplementary material for: Adults vs. neonates: Differentiation of functional connectivity between the basolateral amygdala and occipitotemporal cortex
Source: PLoS One. 2020 Oct 19;15(10):e0237204. doi: 10.1371/journal.pone.0237204 (PMC7571669; doi:10.1371/journal.pone.0237204)
Supplement: S5 Table — t-test results and corresponding p-values comparing mean connectivity between each functional category in adults. (DOCX) [file pone.0237204.s007.docx]

**S5 Table. Connectivity Differences Between Functional Categories in Adults.**

| **Category 1** | **Category 2** | ***t*** | ***p***_HB_ |
| --- | --- | --- | --- |
| Faces | Scenes  Bodies  Objects  Higher Auditory  Primary Auditory  Primary Visual | 3.930  -1.423  -0.348  -2.808  3.3358  7.810 | 0.005  0.928  1.460  0.070  0.023  4.417 x 10^-8^ |
| Scenes | Bodies  Objects  Higher Auditory  Primary Auditory  Primary Visual | -5.340  -4.539  -5.499  -0.164  4.927 | 8.960 x 10^-5^  9.531 x 10^-4^  5.650 x 10^-5^  1.460  2.993 x 10^-4^ |
| Bodies | Objects  Higher Auditory  Primary Auditory  Primary Visual | 1.387  -1.492  4.146  9.027 | 0.814  1.006  0.003  1.199 x 10^-9^ |
| Objects | Higher Auditory  Primary Auditory  Primary Visual | -2.058  3.197  8.283 | 0.371  0.030  1.077 x 10^-8^ |
| Higher Auditory | Primary Auditory  Primary Visual | 5.683  7.000 | 3.429 x 10^-5^  5.362 x 10^-7^ |
| Primary Auditory | Primary Visual | 3.237 | 0.030 |

t-test results and corresponding p-values comparing mean connectivity between each functional category in adults.

Note: p-values are Holm-Bonferroni corrected across all comparisons.
