# Supplementary material for: Adults vs. neonates: Differentiation of functional connectivity between the basolateral amygdala and occipitotemporal cortex
Source: PLoS One. 2020 Oct 19;15(10):e0237204. doi: 10.1371/journal.pone.0237204 (PMC7571669; doi:10.1371/journal.pone.0237204)
Supplement: S6 Table — t-test results and corresponding p-values comparing mean-centered connectivity between adults vs. neonates for each functional category. See Fig 3B in main manuscript. (DOCX) [file pone.0237204.s008.docx]

**S6 Table. Functional Category Connectivity Differences Between Samples.**

| **Category** | ***t*** | ***p***_HB_ |
| --- | --- | --- |
| Faces  Scenes  Bodies  Objects  Higher Auditory  Primary Auditory  Primary Visual | 4.388  -3.216  3.929  3.059  4.740  -2.564  -7.458 | 1.776 x 10^-4^  0.006  7.316 x 10^-4^  0.006  5.664 x 10^-5^  0.012  7.321 x 10^-10^ |

t-test results and corresponding p-values comparing mean-centered connectivity between adults vs. neonates for each functional category. See Fig 3B in main manuscript.

Note: p-values are Holm-Bonferroni corrected.
