# Supplementary material for: Adults vs. neonates: Differentiation of functional connectivity between the basolateral amygdala and occipitotemporal cortex
Source: PLoS One. 2020 Oct 19;15(10):e0237204. doi: 10.1371/journal.pone.0237204 (PMC7571669; doi:10.1371/journal.pone.0237204)
Supplement: S1 File — (PDF) [file pone.0237204.s009.pdf]

# List of Anatomical labels combined to create OTC

## Neonates (from DRAW-EM labels):

- Anterior temporal lobe – medial part
- Anterior temporal lobe – lateral part
- Gyri parahippocampalis et ambiens – anterior part
- Gyri parahippocampalis et ambiens – posterior part
- Superior temporal gyrus – middle part
- Superior temporal gyrus – posterior part
- Medial and inferior temporal gyri – anterior part
- Medial and inferior temporal gyri – posterior part
- Lateral occipitotemporal gyrus – gyrus fusiformis – anterior part
- Lateral occipitotemporal gyrus – gyrus fusiformis – posterior part
- Occipital lobe

## Adults (from aparc+aseg):

- Superior, Middle, and Inferior Temporal cortex
- Banks of the Superior Temporal Sulcus
- Fusiform Gyrus
- Transverse Temporal cortex
- Entorhinal cortex
- Temporal Pole
- Parahippocampal cortex
- Lateral Occipital cortex
- Lingual Gyrus
- Cuneus
- Pericalcarine cortex
