# Supplementary material for: Adults vs. neonates: Differentiation of functional connectivity between the basolateral amygdala and occipitotemporal cortex
Source: PLoS One. 2020 Oct 19;15(10):e0237204. doi: 10.1371/journal.pone.0237204 (PMC7571669; doi:10.1371/journal.pone.0237204)
Supplement: Attachment — Submitted filename: Response to Reviewers.docx [file pone.0237204.s010.docx]

Dear Dr. Hinckley,

Thank you for evaluating our manuscript, “Differentiation of functional connectivity between the basolateral amygdala and occipitotemporal cortex” (PONE-D-20-22317). We are grateful for the positive evaluation, and for the opportunity to revise it for inclusion in PLOS ONE. We would also like to thank each of the reviewers for their time in providing feedback on the manuscript. Below, we have pasted the points raised during the review process (indented), along with our point-by-point responses after each comment (flush left, bolded). We hope the revised version successfully addresses the reviewer’s comments, and that it can be considered suitable for publication,

Sincerely,

Heather Hansen, Jin Li, & Zeynep Saygin

Journal Requirements:

When submitting your revision, we need you to address these additional requirements.

1. Please ensure that your manuscript meets PLOS ONE's style requirements, including those for file naming. The PLOS ONE style templates can be found at

[https://journals.plos.org/plosone/s/file?id=wjVg/PLOSOne_formatting_sample_main_body.pdf](https://urldefense.com/v3/__https:/journals.plos.org/plosone/s/file?id=wjVg*PLOSOne_formatting_sample_main_body.pdf__;Lw!!KGKeukY!kU9xMgPnhQ-C2bdRfYE2AsT35K4LD4w3GWCFMIXL8oGPJJWcblXTwZtroumtjOSHidA$) and

[https://journals.plos.org/plosone/s/file?id=ba62/PLOSOne_formatting_sample_title_authors_affiliations.pdf](https://urldefense.com/v3/__https:/journals.plos.org/plosone/s/file?id=ba62*PLOSOne_formatting_sample_title_authors_affiliations.pdf__;Lw!!KGKeukY!kU9xMgPnhQ-C2bdRfYE2AsT35K4LD4w3GWCFMIXL8oGPJJWcblXTwZtroumt2wU7URg$)

**We have adjusted our figures using the PACE tool to match journal size requirements. We have also made small stylistic changes throughout the manuscript body. This manuscript meets PLOS ONE’s style requirements, to the best of our ability.**

[Note: HTML markup is below. Please do not edit.]

Reviewers' comments:

Reviewer's Responses to Questions

**Comments to the Author**

1. Is the manuscript technically sound, and do the data support the conclusions?

The manuscript must describe a technically sound piece of scientific research with data that supports the conclusions. Experiments must have been conducted rigorously, with appropriate controls, replication, and sample sizes. The conclusions must be drawn appropriately based on the data presented.

Reviewer #1: Yes

Reviewer #2: Yes

2. Has the statistical analysis been performed appropriately and rigorously?

Reviewer #1: Yes

Reviewer #2: Yes

3. Have the authors made all data underlying the findings in their manuscript fully available?

The [PLOS Data policy](https://urldefense.com/v3/__http:/www.plosone.org/static/policies.action*sharing__;Iw!!KGKeukY!kU9xMgPnhQ-C2bdRfYE2AsT35K4LD4w3GWCFMIXL8oGPJJWcblXTwZtroumtoGowvwU$) requires authors to make all data underlying the findings described in their manuscript fully available without restriction, with rare exception (please refer to the Data Availability Statement in the manuscript PDF file). The data should be provided as part of the manuscript or its supporting information, or deposited to a public repository. For example, in addition to summary statistics, the data points behind means, medians and variance measures should be available. If there are restrictions on publicly sharing data—e.g. participant privacy or use of data from a third party—those must be specified.

Reviewer #1: Yes

Reviewer #2: Yes

4. Is the manuscript presented in an intelligible fashion and written in standard English?

PLOS ONE does not copyedit accepted manuscripts, so the language in submitted articles must be clear, correct, and unambiguous. Any typographical or grammatical errors should be corrected at revision, so please note any specific errors here.

Reviewer #1: Yes

Reviewer #2: Yes

**We appreciate these positive evaluations.**

5. Review Comments to the Author

Please use the space provided to explain your answers to the questions above. You may also include additional comments for the author, including concerns about dual publication, research ethics, or publication ethics. (Please upload your review as an attachment if it exceeds 20,000 characters)

Reviewer #1: The paper by Hansen et al. analyzed the functional connectivity of the basolateral region of the amygdala to each of five regions along the rostro-caudal axis of the occipitotemporal cortex in adults and in neonates, using data from the HCP and dHCP databases. The study focuses on the amygdalar nucleus that was found to undergo the most age-related changes in a previous study from the same lab, which is also the nucleus with strong connections to the visual system.

They document a decreasing rostro-caudal gradient of connectivity to the anatomically defined regions, to functional areas along that axis, and to functional categories that could involve structures and areas located in more than one of the anatomical regions. None of these gradients was present in neonates, with the exception to some degree of the anatomical gradient. These results are comparable to a similar gradient found in macaque tracer studies.

The results are clearly presented, and effort was made to make the two sets of data and the anatomical regions in adults and neonates comparable. I acknowledge that my command of the statistics involved in the processing of imaging data is limited, so I am not going to delve into that aspect. But the differences between the two sets of data are statistically robust. There are some intriguing differences -not addressed in the text- in the degree of functional connectivity from the amygdala to some of the regions; for example, to regions 4 & 3. Are they related to any particular area?

**We agree that the differences are intriguing, and are glad the reviewer thinks so too! We examined connectivity to parcels in OTC regions 1-4 to explore these differences, and show that adults have higher connectivity to Face regions (e.g., FFA, STS, OFA), Body regions (e.g., EBA), Object regions (e.g., LO, PFS), and high-level auditory regions (e.g., STG) than do neonates. STG is in region 4, STS and PFS are in region 3, EBA, LO, and FFA are in region 2, and OFA is in region 1 (See Figures 2 and 3). These functional areas seem to be driving the connectivity differences observed in the OTC regions between the two groups. We have now clarified this point explicitly in the text and thank the reviewer for pointing it out (see p. 17).**

The manuscript is clearly written; however, I think the Results section would gain in clarity if a summary sentence was added before the detailed statistics of each set of data.

**To aid in clarity, we have added a summary sentence at the beginning of each interaction paragraph before the detailed statistics. See the revised manuscript (p. 12, 14, 15).**

Minor points:
1. Is the adult template used to define the amygdala subnuclei applicable to neonates?. There is a study that shows differences in size and in the relative area of several amygdalar nuclei in infants as compared to adults (Proc Natl Acad Sci U S A. 2018 Apr 3; 115(14): 3710–3715.

**The adult template used to define the amygdala subnuclei (Saygin et al., 2017) takes each individual’s underlying anatomy into account, so we believe the spatial sensitivity of this template is the best approximation for neonates given that Nissl tissue staining (as was used in the referenced paper) is unavailable in living samples. We additionally did an overlap check (see Figure S1) to make sure, at least, that the basolateral amygdala used here lies within the dHCP-defined amygdala (which it does). We do acknowledge that the nuclei may change through development and have added this point and the referenced study to the Discussion (p.19).**

2. The ventral visual stream is highly immature at birth. Some reference could be made to the maturation timetable of some of the regions depicted in the study.

**The reviewer makes a good point. We have added additional references commenting on the development of cortical selectivity and maturation timetable of the ventral visual stream (see p. 18-19)**

3. When discussing some other work in infants, reference could be made of Biol Psychiatry Cogn Neurosci Neuroimaging. 2019 Jan; 4(1): 62–71., that also studied the amygdala functional connectivity during infancy, including neonates.

**We thank the reviewer for this suggestion! We have incorporated the recommended reference into our Discussion (see p. 18).**

4. In figure 3, A1 seems to be misplaced: It looks as if it is on the frontal lobe, or near the central sulcus.

**The A1 region we use for analyses is located in the superior temporal lobe. We realize the confusion might have arisen given that the text label for A1 on Figure 2 (assuming the reviewer is referring to the figure in which the parcels are displayed) is placed more in the frontal lobe. We have revised this figure to move the A1 text label closer to the orange A1 temporal region to which it refers.**

Reviewer #2: General comments:

In this study was investigated the changes in functional connectivity between the basolateral amygdala and the occipitotemporal cortex that are induced in human brain maturation between neonates and adult subjects. The results suggest that the functional connectivity between these two brain structures is not yet differentiated in neonates.
The results are interesting and will be an important contribution to understand how the emotional circuit matures in the human brain. The authors used a correct experimental design and the manuscript is well written.

Minor concerns:

The following points should be addressed in the discussion section:

1) The functional connectivity between the amygdala and the neocortex somehow tells us how the human brain emotionally translates the world that it lives. So, the authors could suggest how the changes in functional connectivity they found could affect the emotional processing of sensory stimuli that are perceived from the environment. How does the brain of neonates emotionally translate the world? what is lost or possibly gained when the brain matures into adulthood? What your results tell you about these questions.

**We thank the reviewer for this suggestion! We have added commentary on how neonates emotionally translate the world into our discussion (see p.17-18).**

2) The frontal cortex has a fundamental role in the amygdala activity, I understand that the authors did not explore this part of the story in this investigation. However, this is a key piece of the puzzle that should not be excluded when interpreting the results. GABAergic activity is immature in the frontal cortex of neonates, how can this affect the maturation of functional connectivity between basolateral amygdala and occipitotemporal cortex?

**While we agree exploring frontal GABAergic activity would be an interesting follow-up question, it is beyond the scope of the present study (and cannot be assessed with the neuroimaging scans that are currently available from the HCP/dHCP datasets). However, we’ve taken note of this suggestion and addressed this idea as an area of future research in our Discussion (see p. 20).**

3) How can the environment affect neural circuits and induce the functional connectivity changes found in this study?

**Good question! We have added commentary on how the environment might affect neural circuits into our Discussion (see p. 19).**

4) This study was conducted only in women. So, how sex hormones could contribute to the changes in functional connectivity found in this study.

**The study was not conducted only in women. We noted in the Method section that we had 40 neonates and 40 adults, with 15 females in each group. We recognize it would be better practice not to make readers assume the remaining participants are male and have amended the Method section to include counts of both female and male participants.**

6. PLOS authors have the option to publish the peer review history of their article ([what does this mean?](https://urldefense.com/v3/__https:/journals.plos.org/plosone/s/editorial-and-peer-review-process*loc-peer-review-history__;Iw!!KGKeukY!kU9xMgPnhQ-C2bdRfYE2AsT35K4LD4w3GWCFMIXL8oGPJJWcblXTwZtroumtPPnMCMM$)). If published, this will include your full peer review and any attached files.


If you choose “no”, your identity will remain anonymous but your review may still be made public.


**Do you want your identity to be public for this peer review?** For information about this choice, including consent withdrawal, please see our [Privacy Policy](https://urldefense.com/v3/__https:/www.plos.org/privacy-policy__;!!KGKeukY!kU9xMgPnhQ-C2bdRfYE2AsT35K4LD4w3GWCFMIXL8oGPJJWcblXTwZtroumtCO9fVvc$).

Reviewer #1: No

Reviewer #2: **Yes:** Alexies Dagnino-Subiabre

[NOTE: If reviewer comments were submitted as an attachment file, they will be attached to this email and accessible via the submission site. Please log into your account, locate the manuscript record, and check for the action link "View Attachments". If this link does not appear, there are no attachment files.]

While revising your submission, please upload your figure files to the Preflight Analysis and Conversion Engine (PACE) digital diagnostic tool, [https://pacev2.apexcovantage.com/](https://urldefense.com/v3/__https:/pacev2.apexcovantage.com/__;!!KGKeukY!kU9xMgPnhQ-C2bdRfYE2AsT35K4LD4w3GWCFMIXL8oGPJJWcblXTwZtroumtRmzsgsQ$). PACE helps ensure that figures meet PLOS requirements. To use PACE, you must first register as a user. Registration is free. Then, login and navigate to the UPLOAD tab, where you will find detailed instructions on how to use the tool. If you encounter any issues or have any questions when using PACE, please email PLOS at [figures@plos.org](mailto:figures@plos.org). Please note that Supporting Information files do not need this step.
